# Advanced Manufacturing on Nano- and Microscale

**DOI:** 10.3390/nano15110852

**Published:** 2025-06-02

**Authors:** Ruifeng Zhang, Qifeng Ruan, Hao Wang, Yujie Ke

**Affiliations:** 1 Guangdong Provincial Key Laboratory of Semiconductor Optoelectronic Materials and Intelligent Photonic Systems, Harbin Institute of Technology, Shenzhen 518055, China; rf_zhang98@163.com; 2 School of Interdisciplinary Studies, Lingnan University, Tuen Mun, Hong Kong, China; 3 Quantum Science Center of Guangdong-Hong Kong-Macao Greater Bay Area (Guangdong), Shenzhen 518045, China; 4 School of Instrumentation and Optoelectronic Engineering, Beihang University, Beijing 100191, China; 5 Hangzhou International Innovation Institute, Beihang University, Hangzhou 311115, China

## 1. Introduction

Micro- and nanostructures often display unique characteristics that significantly differ from those of bulk materials. The exploration of novel physical principles at these scales has driven the rapid evolution of diverse fabrication strategies. Advanced micro/nanomanufacturing techniques have not only expanded our capacity to probe fundamental physical, chemical, and biological processes, but have also accelerated the integration of miniaturized functionalities into optical, electronic, magnetic, mechanical, and acoustic systems. These fabrication methods, broadly classified into additive and subtractive approaches, encompass technologies such as electron beam lithography, focused ion beam processing, photolithography, direct laser writing (DLW) [1], nanoimprint lithography [2], solution-based synthesis [3], and self-assembly [3,4]. The material repertoire has expanded from conventional substrates such as silicon, metals, and polymers to include biomaterials and emerging two-dimensional materials [2,4,5]. The resulting structures span from intricate 2D/3D architectures to reconfigurable systems incorporating time-dependent behaviors [6]. Furthermore, the integration of multiple materials and processes into hybrid fabrication schemes has shown great promise in addressing the dual demands of precision and scalability, paving the way toward sustainable and cost-effective manufacturing for industrial-scale deployment.

Recent advancements in advanced materials and fabrication techniques have enabled transformative innovations across multiple domains. Bio-inspired manufacturing, such as nacre-like chitosan/CaCO₃ composites synthesized through room-temperature mineralization, demonstrates exceptional mechanical properties and underwater superoleophobicity [7]. Laser-based technologies, including femtosecond laser ablation of perovskites and optothermal spatiotemporal control of phase-separated liquids, achieve subwavelength patterning and functional complexity for photonic and biomedical applications [8,9]. Breakthroughs in additive manufacturing encompass enzyme-responsive hydrogel lasers for anti-counterfeiting, hierarchically self-assembled chiral microtoroids for light harvesting, and two-photon-polymerized luminescent microarchitectures for quantum sensing [10,11,12]. Nanomaterial synthesis innovations such as thermally activated ligand chemistry and direct optical patterning of perovskites enhance optoelectronic integration, while scalable microreactor systems enable green synthesis of nanoparticles and metal–organic frameworks [13,14,15]. Emerging interdisciplinary applications include functional textiles with glucose-sensing nanopatterns, artificial intelligence-driven material discovery for energy storage, and MXene/polyimide aerogels for ultra-broadband microwave absorption [16,17,18].

## 2. An Overview of Published Articles

This Special Issue highlights eight pioneering studies that address critical challenges in advanced manufacturing. These include the fabrication of diffractive optical elements (DOEs) for next-generation optics, efficient light coupling in photonic systems, robust fiber sensors for harsh environments, advanced magnetic composites, microfluidic synthesis of nanoparticles, high-entropy alloy coatings, and reproducible surface-enhanced Raman scattering (SERS) platforms. Each study combines novel fabrication techniques, material optimization, and rigorous characterization to push the boundaries of performance, reliability, and scalability.

Contribution 1. Diffractive Optical Element Microfabrication

Dai et al. [19] introduced a photolithography-based method to fabricate DOEs on silicon and sapphire substrates, achieving precise control over refractive index and thickness. Using 3 × 3 and 3 × 5 beam splitter designs, the study demonstrated a remarkable alignment between simulations and experimental results, with discrepancies as low as 0.53% (silicon) and 0.57% (sapphire). The scalability and precision of this technique make it promising for semiconductor manufacturing and optical systems such as light detection and ranging (LiDAR) and augmented reality (AR) and virtual reality (VR).

Contribution 2. Efficient Mode Conversion via Tapered Fibers

Wu et al. [20] fabricated a tapered fiber via DLW-enabled efficient light transition from a single-mode fiber to a subwavelength microfiber. Simulations guided the design, achieving 86% theoretical transmittance. Experiments achieved 77% transmittance at 1550 nm, compressing the mode area from 38 μm^2^ to 0.47 μm^2^ over 150 μm. This advancement supports fiber-to-chip integration for photonic circuits and quantum technologies.

Contribution 3. Fiber Bragg Gratings with Femtosecond Laser-Inscribed Microvoids

Sosa et al. [21] engineered Type III fiber Bragg gratings using femtosecond lasers to create periodic microvoids in fiber cores. High-resolution microscopy revealed densified shells around the voids, which maintained structural integrity up to 1250 °C. These findings aid in optimizing fiber sensors for extreme environments such as aerospace and energy systems.

Contribution 4. Magnetic Composites for Soft Magnetic Applications

Mesaros et al. [22] synthesized Fe@Fe_3_O_4_/ZnFe_2_O_4_ soft magnetic composites (SMCs) via hybrid cold sintering/spark plasma sintering. The composite exhibited a saturation induction of 0.8 T and coercivity of 590 A/m. While AC losses dominated at high frequencies due to eddy currents, the DC performance matched state-of-the-art SMCs, suggesting utility in power electronics and motors.

Contribution 5. Microfluidic Synthesis of Calcium Carbonate Nanoparticles

Reznik et al. [23] introduced a two-phase microfluidic method to produce CaCO_3_ nanoparticles (50 nm, 86–99% vaterite) with superior size control compared to one-phase systems. Morphological and structural analysis and dynamic light scattering validated the approach, highlighting potential for drug delivery, coatings, and catalysis.

Contribution 6. High-Entropy Alloy Coatings for Enhanced Surface Properties

Ren et al. [24] fabricated FeCoNiCrMo0.2 coatings with WC and CeO_2_ additives via laser cladding onto 316L stainless steel. The WC-enhanced coating showed high hardness but uneven particle distribution. Adding CeO_2_ refined grains, reduced porosity, and improved corrosion resistance, yielding a balanced composite with extended workpiece lifespan.

Contribution 7. SERS Platform via Nanoimprint Lithography

Milenko et al. [25] used UV-nanoimprint lithography (UV-NIL) to create large-area, ordered C-shaped gold nanostructures for SERS spectroscopy techniques. Avoiding silicon etching reduced costs and improved reproducibility. The platform detected Rhodamine 6G with high sensitivity, offering a scalable solution for biosensing and environmental monitoring.

Contribution 8. Gradient Interfaces for Thermal Stress Mitigation via Co-Deposition

Zhang et al. [26] presented an innovative approach for fabricating high-performance SiC whisker-reinforced copper matrix functionally graded materials (FGMs) using co-electrodeposition and numerical simulation. The FGMs were designed to serve as an interface layer between Cu and Si in high-power electronic devices, addressing thermal stress caused by mismatched coefficients of thermal expansion. The gradient structure effectively mitigates thermal stress, enhances mechanical robustness, and improves interfacial reliability in electronic packaging, offering a scalable, low-temperature manufacturing solution for advanced thermal management in high-power devices.

## 3. Conclusions

Collectively, these studies exemplify cutting-edge innovations in optics, materials, and nanotechnology for advanced manufacturing, offering tailored solutions for energy, healthcare, and beyond. The DOE fabrication method and tapered fiber design advance optical systems for AR/VR and photonics. Fiber Bragg gratings and magnetic composites address durability challenges in harsh environments and power applications. Microfluidic synthesis and high-entropy alloy coatings optimize material properties for industrial and biomedical uses, while the UV-NIL SERS platform enhances sensing reproducibility.

These breakthroughs highlight interdisciplinary approaches to solving technical bottlenecks, paving the way for scalable, high-performance technologies in fields such as healthcare, energy, and telecommunications. Challenges in scalability, material compatibility, and artificial intelligence integration remain, but the future promises unprecedented functional versatility and sustainability in materials science. Future work can focus on refining fabrication scalability, material stability, and integration into real-world systems.
